# Autoimmune glial fibrillary acidic protein astrocytopathy manifesting as subacute meningoencephalitis with descending myelitis: a case report

**DOI:** 10.1186/s12883-020-02021-7

**Published:** 2020-12-10

**Authors:** Han Wang, Jerome H. Chin, Bo-yan Fang, Xi Chen, Ai-lin Zhao, Hai-tao Ren, Hong-zhi Guan

**Affiliations:** 1 Department of Neurology, Peking Union Medical College Hospital, Chinese Academy of Medical Sciences, Beijing, 100730 China; 2 Department of Neurology, NYU Langone Health, New York, NY USA; 3 Department of Neurology, Beijing Rehabilitation Hospital Affiliated to Capital Medical University, Beijing, China; 4 Department of Neurology, Tonghua Central Hospital, Jilin, China; 5 Chinese Academy of Medical Sciences and Peking Union Medical College, Beijing, China

**Keywords:** GFAP, Encephalitis, Meningitis, Myelitis, Autoimmune

## Abstract

**Background:**

Glial fibrillary acidic protein (GFAP) autoimmune astrocytopathy is characterized by GFAP autoantibody positive encephalitis, meningoencephalitis or meningoencephalomyelitis. The initial clinical presentation may be similar to central nervous system infections making early diagnosis challenging.

**Case presentation:**

A Chinese female patient presented with subacute meningitis with symptoms of headache, vomiting, and fever. Cerebrospinal fluid (CSF) analysis showed monocytic pleocytosis, elevated protein level, low glucose level, and negative basic microbiological studies including Xpert MTB/RIF. Brain magnetic resonance imaging (MRI) showed bilateral cerebral cortical and white matter hyperintensities on FLAIR sequences. The patient was diagnosed with possible tuberculous meningitis and started on anti-tuberculosis therapy (ATT). Three months later, the patient developed cervical myelopathy and encephalopathy with persistent CSF pleocytosis. Five months later, tissue-based and cell-based assays demonstrated GFAP antibodies in blood and CSF. Her symptoms improved with repeated administration of intravenous immunoglobulin (IVIG) and corticosteroids. One-and-a-half -year follow-up showed neither clinical progression nor relapses.

**Conclusions:**

Anti-GFAP astrocytopathy should be included in the differential diagnosis of patients who present with subacute meningitis with negative microbiological studies and a progressive clinical course including encephalitis and/or myelitis.

## Background

Autoimmune astrocytopathy with antibodies to glial fibrillary acidic protein (GFAP) was first described in 2016 [1]. The predominant clinical syndrome is meningoencephalo-myelitis or a limited form of the same, i.e. meningitis, encephalitis, and/or myelitis [1–7]. The biomarker of this disorder is auto-antibody in CSF against GFAPα, the predominant intermediate filament protein in adult astrocytes, detected by cell-based testing. Since GFAP is an intracellular antigen, it is unknown if another target is responsible for the immuno-pathogenesis of the clinical manifestations of this autoimmune neurological disorder. Case reports and case series have described associations of anti-GFAP astrocyopathy with neoplasms and prodromal infectious symptoms [6]. Here we report a Chinese patient who developed a subacute febrile meningitis and was treated for tuberculosis for 9 months prior to the diagnosis of anti-GFAP astrocytopathy with clinical involvement of brain and spinal cord documented by neuroimaging.

## Case presentation

**Fig. 1:**
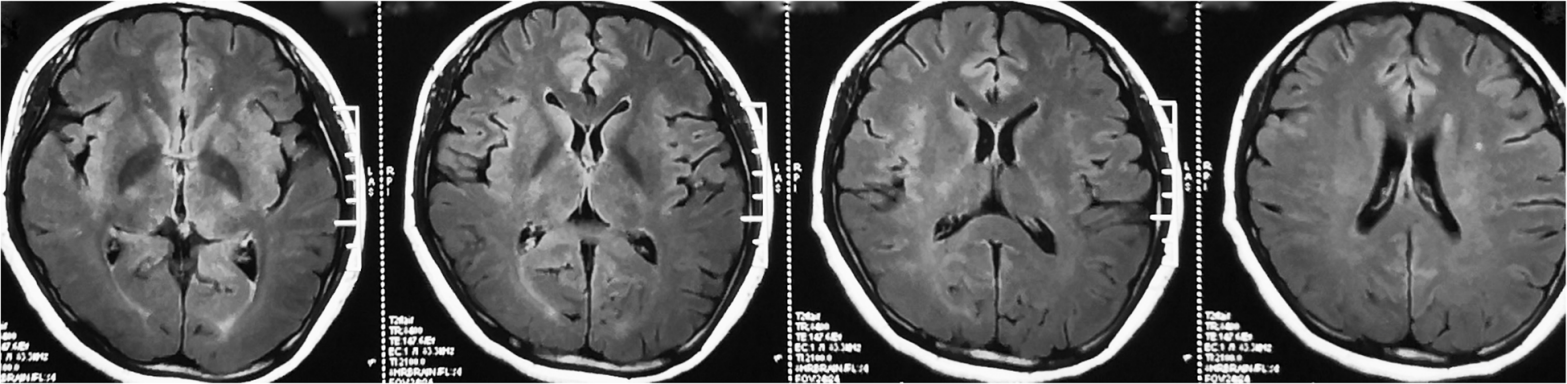
Axial T2 FLAIR sequence MRI showing bilateral cortical and subcortical hyperintensities

A 28-year-old female was admitted with fever (39.4 °C), headache, vomiting, and cough for 1 week. Medical history was notable for chronic hepatitis B. Chest CT was normal. Brain MRI showed hyperintensities on T2 FLAIR sequences in bilateral cortical and subcortical regions (Fig. 1). No abnormal enhancement or hydrocephalus was seen. Cerebrospinal fluid (CSF) analysis showed glucose 2.52 mmol/L, protein 1.76 g/L, and white blood cells 300 × 10^6^/L (80% mononuclear cells). Acid fast stain, India Ink preparation, and bacterial culture were negative. She experienced one convulsion. Gancyclovir was administered for 3 days. Possible tuberculous meningitis was diagnosed and anti-tuberculosis therapy (ATT) was started with oral rifampicin, pyrazinamide, and ethambutol. Isoniazid and dexamethasone were administered intrathecally every 2–6 days for 1 month. CSF was sampled six times during intrathecal treatments and showed persistent pleocytosis with white blood cells of 80 to 394 cells × 10^6^/L (80–90% mononuclear cells). Ethambutol was withdrawn due to blurred vision and replaced with levofloxacin. Her fever and headaches improved but tremor developed in both hands.

**Fig. 2:**
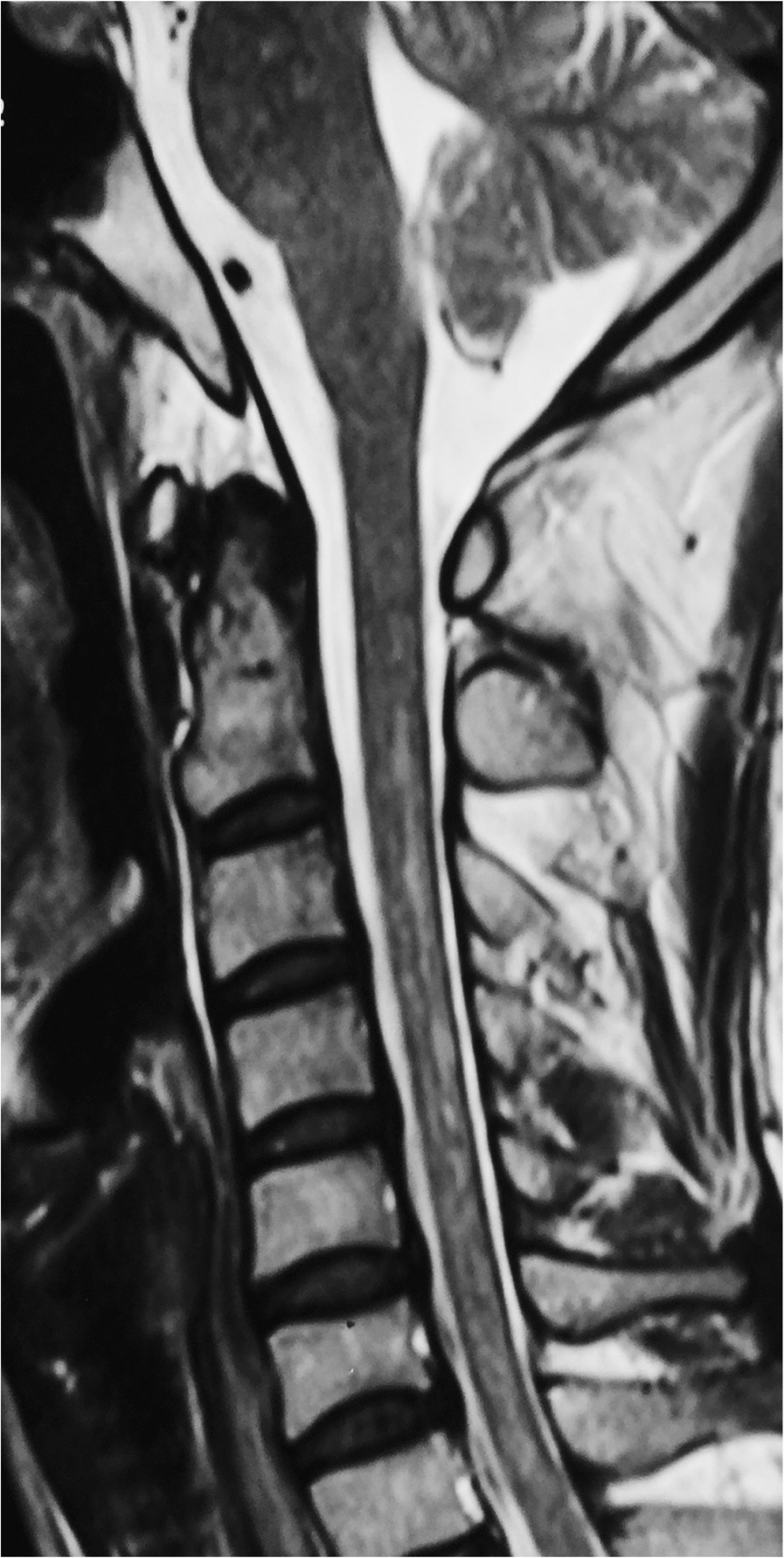
Sagittal T2 sequence MRI showing patchy hyperintensities throughout the cervical spinal cord

**Fig. 3:**
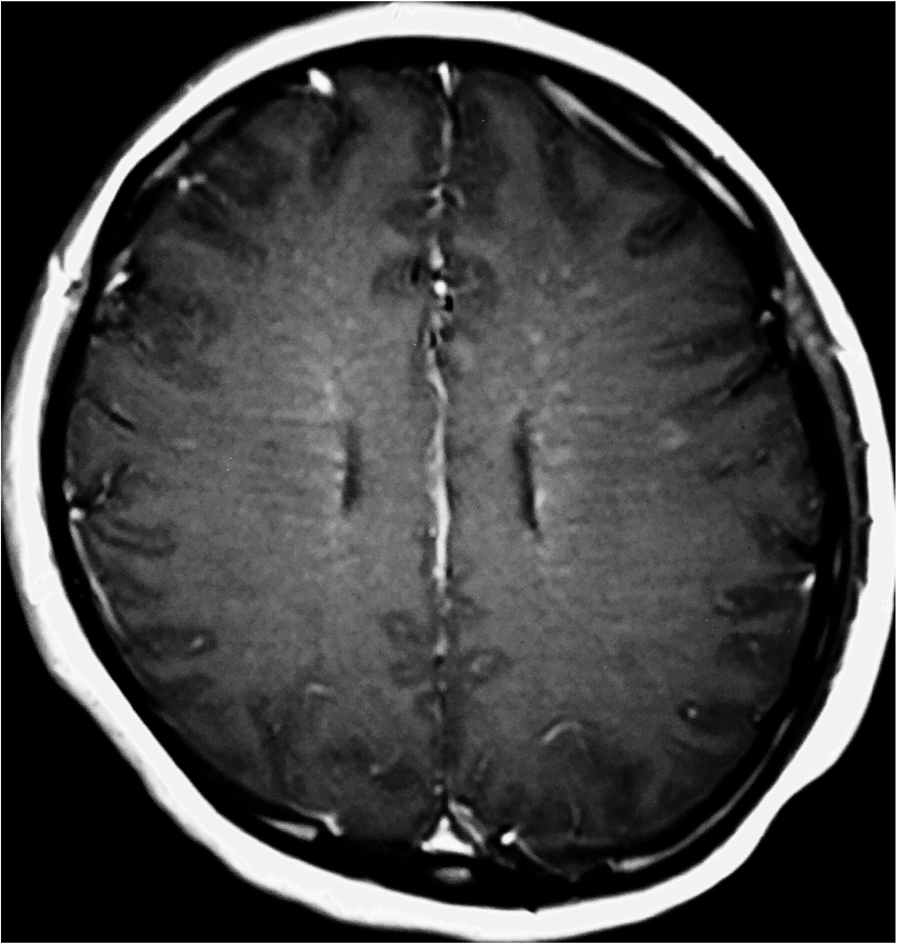
Axial T1 post-contrast sequence MRI showing a radial pattern of linear periventricular post-gadolinium enhancement

**Fig. 4:**
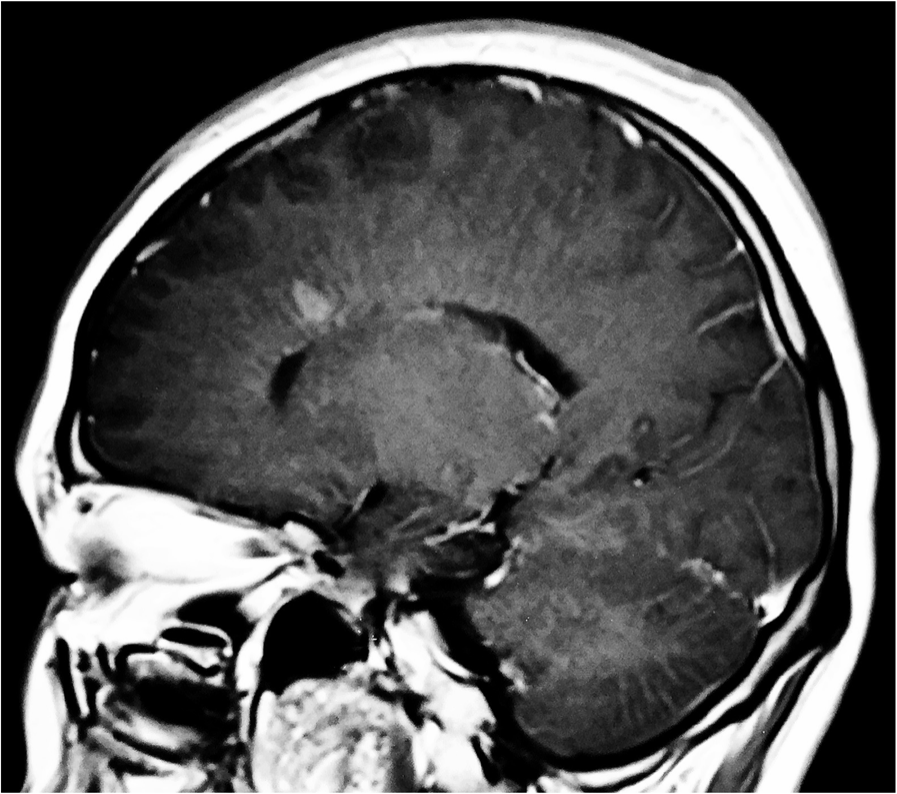
Sagittal T1 post-contrast sequence MRI showing a radial pattern of linear periventricular post-gadolinium enhancement

Three months later, she developed back pain, leg weakness, memory loss, irritability, and confusion. These symptoms progressed and eventually she could not walk independently. Cervical MRI demonstrated patchy hyperintensities in the spinal cord extending from C2-C7 (Fig. 2). Brain MRI demonstrated linear and radial gadolinium enhancement extending perpendicular to the lateral ventricles (Figs. 3 and 4). CSF analysis showed glucose 2.32 mmol/L, protein 1.09 g/L, and white blood cells 52 × 10^6^/L (90% mononuclear cells). Testing of CSF for *Mycobacterium tuberculosis* (MTB) by nucleic acid amplification testing (Xpert MTB/RIF) and culture (MGIT) were both negative. ATT was adjusted to moxifloxacin, amikacin, isoniazid, rifampicin, and pyrazinamide. Intravenous immunoglobulin (IVIG) was administered (25 g/day × 5 days) with improvement of her symptoms. Methylprednisolone was given intravenously (1 g/day × 3 days, 500 mg/day × 3 days) without further improvement.

She was referred to our hospital 5 months later. She had weakness of the right leg, hyperactive deep tendon reflexes, bilateral Babinski signs, unsteady gait, limb incoordination, and hand tremor. ATT consisted of rifampicin, pyrazinamide, and moxifloxacin. CSF analysis showed glucose 2.7 mmol/L, protein 0.89 g/L, white blood cells 14 × 10^6^/L (86% mononuclear cells), and positive oligoclonal bands. Blood tests for TSH, Free T4, T3, B12, ESR, CRP were normal. Serum tests for infection including HIV 1/2 antibody, HSV1 IgM, HSV2 IgM and IgG, CMV IgM, EBV DNA, HCV, cryptococcal antigen, and TPPA were negative. Tests for autoimmune disease (ANA, anti-DS DNA, anti-RNP, anti-SSA/SSB, anti-Scl-70, anti-Jo-1, anti-Sm, ANCA, anti-TPO) and neoplasm (CA 242, SCC Ag, AFP, CEA, CA19–9, CA125, CA72–4, CA15–3, Cyfra 21–1) were negative. The following serum autoimmune encephalitis antibodies were negative: Hu, Yo, Ri, Amphiphysin, Ma2/Ta, CV2/CRMP5, AQP4, NMDA, AMPA1, AMPA2, GABAB, CASPR2, LGI1, GAD65.

**Fig. 5:**
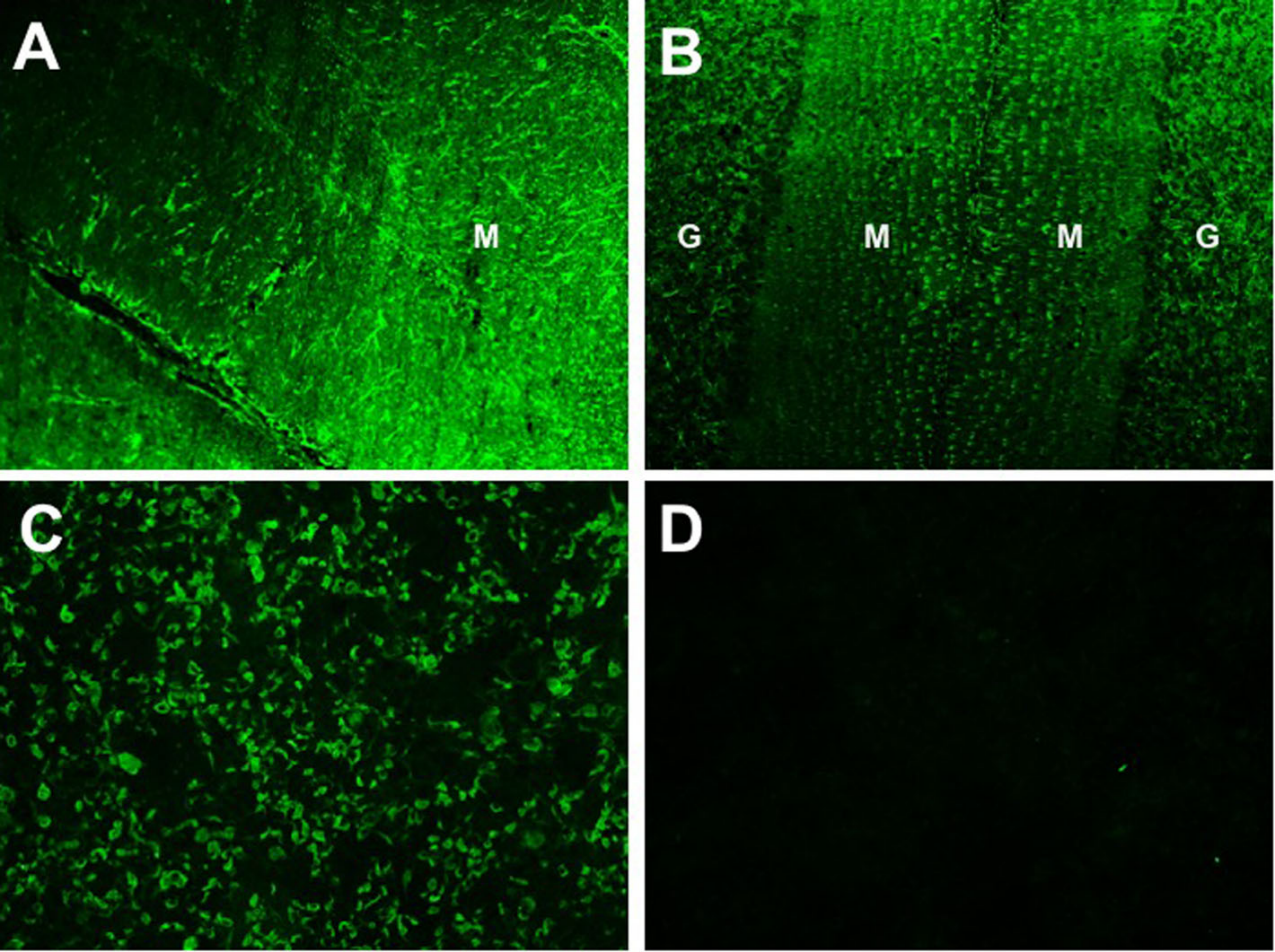
Tissue-based and cell-based indirect immunofluorescence assays demonstrating GFAPα–immunoglobulin G (IgG) in CSF from our patient**.** Astrocytic staining of GFAPα- IgG is prominent in the molecular layer (M) of hippocampus (Panel **a**) and the granular layer (G) of cerebellum (Panel **b**). The short radial staining pattern of Bergmann glia in the molecular layer (M) of cerebellum (Panel **b**) is a characteristic feature of GFAPα - IgG immunoreactivity. CSF is reactive with commercial GFAPα -transfected cells (Shaanxi MYBiotech Co. Ltd., China (Panel **c**). CSF from idiopathic intracranial hypertension patients served as negative controls (Panel **d**). (Magnification times: 200X)

After reviewing of the brain MRI studies from the referring hospital, the linear perivascular radial gadolinium enhancement patterns (Figs. 3 and 4) made us suspect anti-GFAP astrocyopathy. Tissue-based and cell-based assays for GFAP antibodies were sequentially tested and antibodies were detected in blood and CSF. The GFAP antibody titers in serum and CSF were 1:32 and 1:100 respectively (Fig. 5). Repeat brain MRI revealed persistent but subtle radial periventricular enhancement (image not shown). IVIG (25 g/day × 5 days) and prednisone (30 mg daily) were administered. Her leg weakness improved and she could ambulate without assistance at discharge. Prednisone was continued and tapered over 6 months. She has been followed for one and a half year without progression or relapses.

## Discussion and conclusions

Anti-GFAP astrocyopathy is an autoimmune disorder with a broad spectrum of neurological presentations [1–5]. Fever, headache, and meningeal symptoms are frequently reported as initial symptoms and some patients have prodromal influenza-like symptoms. This has raised speculation that certain cases of autoimmune GFAP astrocyopathy may be induced by an antecedent viral or bacterial infection. Our patient was diagnosed with tuberculous meningitis without microbiological confirmation and treated with ATT and dexamethasone. Her early symptoms improved but she developed new and progressive neurological symptoms including cognitive and motor deficits. CSF analyses demonstrated persistent pleocytosis and elevated protein levels for 9 months which has not been previously reported to our knowledge in patients with anti-GFAP astrocytopathy. In a case series of fourteen patients in Japan, CSF pleocytosis was detected in one patient up to 6 months [3].

Patients with subacute meningitis and negative routine microbiological studies of CSF for non-mycobacterial bacteria and fungi are often treated empirically with ATT since no microbiological test including cultures can rule out tuberculous meningitis [8]. Kimura et al. [3] reported five of fourteen patients with anti-GFAP astrocytopathy who were initially diagnosed with tuberculosis meningitis and treated with ATT. Iorio et al. [4] described one patient that was initially diagnosed with tuberculous meningitis. All of these cases had negative polymerase chain reaction testing and culture for MTB from CSF specimens. Notably, Kimura et al. [3] reported transient elevations of adenosine deaminase (ADA; cutoff value 10 IU/L) in the CSF of most of their patients with anti-GFAP astrocytopathy. ADA is a non-specific biomarker that can be elevated in tuberculous meningitis [8]. We did not measure ADA levels in the CSF of our patient.

The differential diagnosis of meningoencephalomyelitis is broad and includes infectious, post-infectious, and autoimmune diseases of the central nervous system. Coexisting neural autoantibodies have been reported in some patients with anti-GFAP astrocytopathy including antibodies to NMDA receptors, GABA-A receptors, and aquaporin-4 [2–4]. Brain and cervical MRI studies in our patient demonstrated abnormalities reported in cases of anti-GFAP astrocytopathy [7] which led to our search for and discovery of antibodies to GFAP in the blood and CSF. Although not specific for autoimmune encephalitis [2], linear perivascular radial gadolinium enhancement extending outwards from the lateral ventricles was seen in our patient and has been reported in case series of patients with anti-GFAP astrocytopathy from the U.S. [2], Japan [3], and China [5] at frequencies of 53, 28.6, and 42.1%, respectively. Long et al. [5] described longitudinally extensive spinal cord lesions on MRI in 11 out of 16 patients and we found similar findings in our patient.

Acute treatments for anti-GFAP astrocytopathy include intravenous methylprednisolone (IVMP), intravenous immunoglobulin (IVIG), intravenous dexamethasone, and plasma exchange with variable responses [2–5]. Three groups [2–4] reported improvements in most of their patients with immunotherapy whereas Long et al. [5] stated, “Most of our patients did not respond very well to routine IVMP and IVIG therapy during the acute stage or long-term treatment with oral steroids and immunosuppressants”. Our patient improved after two separate courses of IVIG. She received one course of IVMP after the first course of IVIG without additional benefit over her positive response to IVIG.

In summary, anti-GFAP astrocytopathy should be considered in the differential diagnosis for patients who present with subacute meningitis and negative microbiological studies for bacteria, mycobacteria, fungi and viruses. A worsening and/or progressive neurological course, including symptoms and signs of encephalopathy and myelopathy, and persistent CSF pleocytosis despite antimicrobial therapy, e.g. ATT, should prompt testing for anti-GFAP antibodies and other neural autoantibodies which may coexist. Whether infections including tuberculous meningitis could have a pathogenic role in the development of anti-GFAP astrocytopathy in certain cases requires further research. Although consensus treatment guidelines are not available, IVMP, IVIG, and plasma exchange may produce improvement in some, but not all patients.

## Data Availability

All data generated or analyzed during this study are included in this published article.

## Abbreviations

GFAP: Glial fibrillary acidic protein
ATT: Anti-tuberculosis therapy
CSF: Cerebrospinal fluid
MTB: *Mycobacterium tuberculosis*
MRI: Magnetic resonance imaging
IVMP: Intravenous methylprednisolone
IVIG: Intravenous immunoglobulin
